# Recurrent bilateral *Mycobacterium bovis* necrotizing epididymitis: a case report

**DOI:** 10.1186/s13104-018-3426-2

**Published:** 2018-05-18

**Authors:** Simon Grandjean-Lapierre, Simon-Djamel Thiberville, Mustapha Fellag, Christophe Eghazarian, Feriel Bouzid, Christina Gavril, Michel Drancourt

**Affiliations:** 1 Aix-Marseille Univ, IRD, MEPHI, IHU Méditerranée Infection, 19-21 Boulevard Jean Moulin, 13005 Marseille, France; 2 Centre Hospitalier Louis Raffalli, 04100 Manosque, France; 3 Centre Hospitalier du Pays d’Aix, 13616 Aix en Provence, France

**Keywords:** *Mycobacterium bovis*, Zoonosis, Epididymitis

## Abstract

**Background:**

*Mycobacterium bovis* causing tuberculosis in animals is responsible for zoonotic tuberculosis in patients. Veterinary control measures and milk pasteurization has led to a significant decrease in human cases of *M. bovis* infections in developed countries.

**Case presentation:**

We diagnosed recurrent *M. bovis* epididymitis in a 63-year old Caucasian man without any signs of pulmonary or disseminated disease. Relevant epidemiological expositions included camel milk drinking during prolonged travels in Niger, prior to initial clinical manifestations. The diagnosis was firmly established by mass spectrometry and DNA sequencing on epididymis surgical biopsy specimens. We detail therapeutic management which included surgical epididymectomy and hydrocele repair.

**Conclusion:**

As for other *M. tuberculosis* complex species, the genitourinary tract represents a frequent site of secondary dissemination and latency for *M. bovis*. Isolated epididymis infection is a newly documented manifestation of *M. bovis* disease.

## Background

*Mycobacterium bovis*, a member of the *Mycobacterium tuberculosis* complex is responsible for bovine-type tuberculosis [1]. Animal to human transmission mainly occurs through airborne route or ingestion of unpasteurized milk. *M. bovis* attenuated Bacille Calmette-Guérin (BCG) strain vaccination campaigns together with milk pasteurization and better veterinary control measures has led to a decrease in the number of human *M. bovis* infections [2]. Sporadic cases of human *M. bovis* suspected zoonotic acquisition are still reported particularly in some developing countries [3]. Also, re-emergence in human populations has been notified in some developed countries still affected by bovine tuberculosis [4].

*Mycobacterium tuberculosis* complex genitourinary infections represented 6.5% of all extra-pulmonary tuberculosis declared cases in the United States between 1993 and 2006 [5]. Although tuberculous epididymitis is a well described clinical entity, no case has ever been documented with *M. bovis* as the causative agent [6]. We here report the first such case in a 63-year old man with recurrent bilateral *M. bovis* granulomatous epididymitis requiring repeated surgical management.

## Case presentation

In 2016, a 63-year-old Caucasian French-born retired man was referred to our institution with a 3-month history of left testicular pain and swelling. He was afebrile and in no apparent distress. Detailed clinical evaluation revealed a tender and swollen left epididymis with associated hydrocele but without torsion, focal testicular mass, purulent discharge or pain on digital rectal examination. He was in a long-term exclusive heterosexual relationship and reported no active risk factors or past episodes of sexually transmitted diseases. Standard bacterial urine cultures following prostate massage were sterile. *Chlamydia trachomatis* and *Neisseria gonorrhea* polymerase chain reaction assays performed on first-void urine were negative for both him and his sexual partner. Syphilis and HIV serology were also non-reactive.

**Fig. 1:**
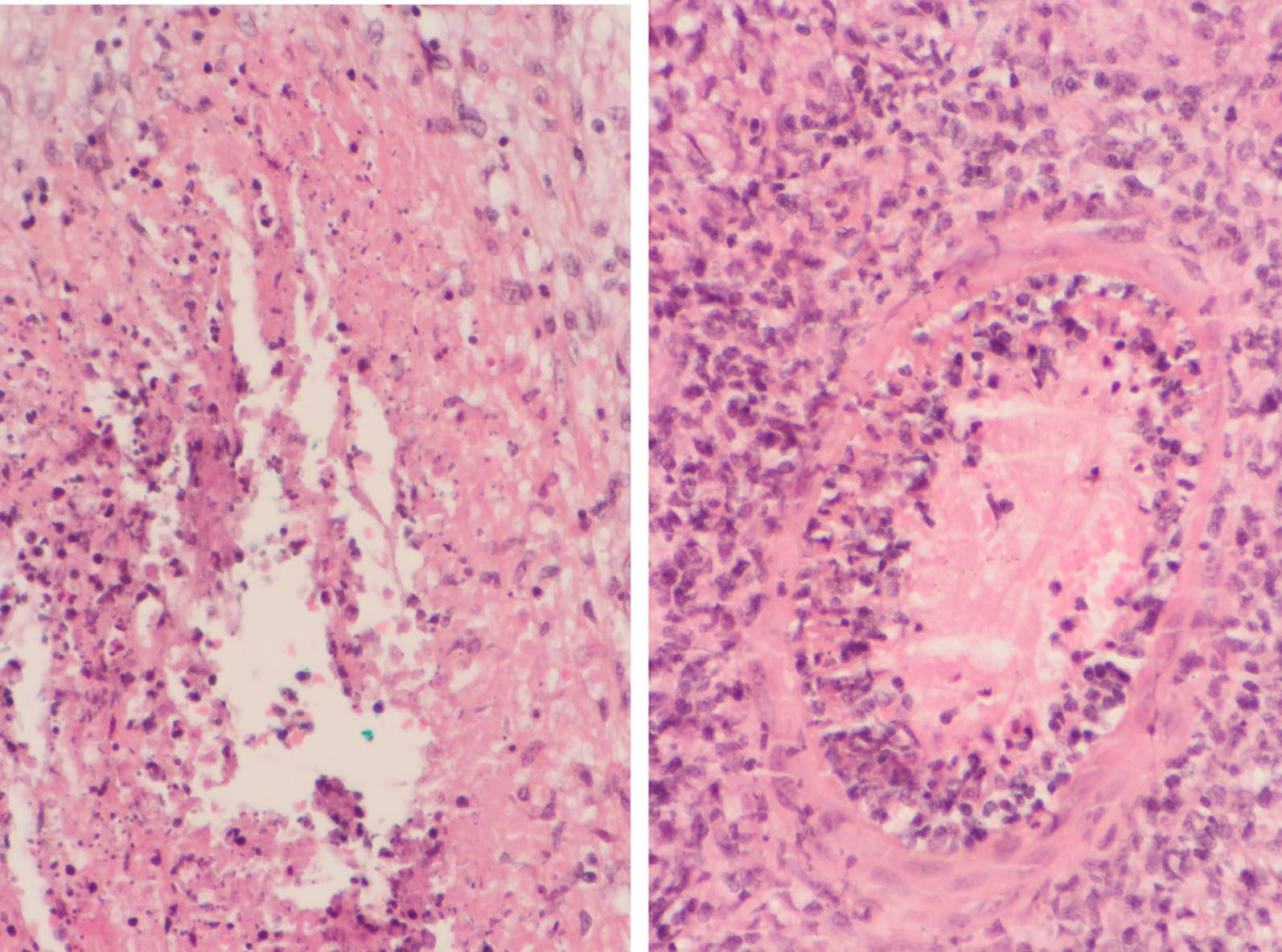
Histopathologic findings on surgical biopsy specimens showing necrotic and caseous abscess lesions (left) and peri-arteriolar tropism of necrotizing and fibrinoid inflammation (right)

Past medical history was unrevealing except for a similar episode which had occurred 6 years before and for which a right epididymectomy had been performed. At that time, results of the histological investigation had shown epididymitis with caseo follicular lesion whereas standard bacterial and mycobacterial culture had failed to identify a specific etiology. A surgical left epididymectomy and hydrocele repair was performed and per-operatory examination revealed pachy-vaginalitis and sub-acute epididymitis with multiple caseous purulent collections. Testes were macroscopically normal. On histological examination, necrotizing and granulomatous epididymitis with caseous abscesses were described (Fig. 1) but periodic acid Schiff and Ziehl stainings showed no microorganisms. The patient was therefore empirically treated for sexually transmitted diseases and bacterial epididymitis with initial intravenous ceftriaxone and oral azithromycin followed by a 3-week course of oral ciprofloxacin.

Three weeks following surgical intervention, mycobacterial liquid medium cultures came back positive. While awaiting final identification and anti-mycobacterial susceptibility testing, a standard anti-tuberculous therapy including isoniazid, rifampin, pyrazinamide and ethambutol was initiated. The isolate was subsequently identified as *M. bovis*, a mycobacterial species known to be intrinsically resistant to the first-line anti tuberculous drug pyrazinamide [7]. Identification to the species level was confirmed using partial 16S rRNA gene (primer fD1: 5′-AGAGTTTGATCCTGGCTCAG-3′; and primer rP2: 5′-ACGGCTACCTTGTTACG ACTT-3′) and partial *rpoB* gene (primer MycoF: 5′-GGCAAGGTCACCCCGAAGGG-3′; primer MycoR: 5′-AGCGGCTGCTGGGTGATCATC-3′) sequencing and mass spectrometry per local procedures [8, 9]. Differentiation between this clinical isolate and the attenuated *M. bovis* BCG strain was confirmed with subsequent multiplex polymerase chain reaction (PCR) assay [10]. Antimycobacterial susceptibility testing showed the isolate to be pan-susceptible to first-line anti-tuberculous drugs other than pyrazinamide and therapy was therefore modified for isoniazid and rifampin alone. Complementary investigation including mycobacterial blood cultures and a whole-body computerized tomodensitometry was also performed and showed no sign of concomitant pulmonary or disseminated disease.

*Mycobacterium bovis* is a recognized zoonotic disease of which genito-urinary infection presentations are rare. Therefore, potential epidemiologic expositions were further detailed. The patient reported numerous prolonged travels in north and sub Saharan Africa in the 80’s and 90’s. During these stays he was in close contacts with cattle and he regularly ingested camel milk in the Niger desert as Touareg habit. He had never received *M. bovis* BCG intravesical irrigations or anti-tuberculous vaccine and denied having ever had sexual relations with animals.

The patient stopped antimycobacterial therapy against medical advice 2 weeks after initiation and still refuses to be treated. Upon submission of this manuscript, 1 year after the initial diagnosis, no local relapse had occurred and urine control mycobacterial culture was negative.

## Discussions and conclusions

*Mycobacterium bovis* is a global zoonotic pathogen. Human infections are almost always acquired by ingestion of unpasteurized dairy products or aerosol inhalation from infected animals [2]. Although cattle are the most frequent source of human infections, other farm and wildlife animals have been shown to be susceptible to *M. bovis* [11]. *M. bovis* survives in the environment and soil reservoirs were proposed as an hypothesis to explain encountered difficulties in bovine-tuberculosis eradication campaigns [12]. Human to human transmission of *M. bovis* appears to be anecdotal [13], what raises the unanswered question of relative virulence of *M. bovis* and *M. tuberculosis* in human beings.

Despite successful bovine tuberculosis eradication programs and more systematic milk pasteurization, human infections still occur. Surveys from low tuberculosis incidence countries where disease had been eradicated in cattle reported a total of 459 human cases over a 20-year period [11]. Among these, the relative proportion of primary infections and late reactivations can’t be established with precision although it is clear that *M. bovis* has the ability to enter dormancy and thus persisting for extended periods of time in individuals similarly to other members of the *M. tuberculosis* complex [14]. This phenomenon could account for this persistent epidemiology in humans. These same surveys estimated that genitourinary infections accounted for 12–53% of human *M. bovis* infections [11]. Kidney, bladder and urinary tract infections mainly account for this epidemiology. A detailed literature review revealed only one previous suspected case of *M. bovis* associated scrotal abscess and granulomatous epididymitis [15]. In this previous study, diagnosis was made on the basis compatible epididymis histopathologic findings concomitant with pulmonary cavitary disease and *M. bovis* positive sputum culture. Although the authors could not confirm the presence of *M. bovis* by culture or molecular biology in an epididymis specimen, this case presentation is compatible with pulmonary infection and secondary testicular dissemination. Oppositely our patient had no signs of active pulmonary or disseminated disease which argues for epididymis focalized reactivation. Although it could not be confirmed retrospectively by microbiologic or histopathologic analyses, our patient had most likely suffered from contralateral *M. bovis* epididymitis 6 years prior to the episode here described. At that time, surgical epididymitis was performed and the patient received no anti-tuberculous drug therapy. These elements reinforce the notion that the genitourinary tract is a frequent sanctuary for *M. tuberculosis* complex bacilli. Table 1 compares the epidemiologic, clinical and therapeutic features of these two discussed cases.

**Table 1:** Reported *Mycobacterium bovis* associated epididymitis

	Age	Sex	Genital disease	Distant infection	Epidemiologic exposition	Treatment		Outcome
						Anti-tuberculous therapy	Surgical management	
Mateos Colino et al. [15]	73	M	Epididymitis scrotal abscess	Lung cavitary lesion	Cattle	Isoniazid + rifampin 12 months	Scrotal drainage	Chronic fistulation
This case	63	M	Epididymitis hydrocele	None	Camel milk	None (refused by patient)	Epididymectomy scrotal drainage	Ongoing treatment

From the microbiology laboratory perspective, the attenuated form of *M. bovis*, Bacille Calmette-Guérin (BCG), can be misidentified as wild-type *M. bovis* using first-line conventional phenotypic and molecular assays. Moreover, *M. bovis* BCG is still widely used in the treatment of bladder cancer and multiple cases of the genitourinary infections following intravesical therapy have been reported [16, 17]. We identified this *M. bovis* clinical isolate using mass spectrometry and *rpoB* gene DNA sequencing [8, 9]. Indeed, alignment of 16S rRNA and *rpo*B gene sequences yielded the same result with 99% sequence similarity with the reference *M. bovis* AF2122/97 (GenBank LT708304.1). A two-step, multiplex PCR was also performed to further distinguish between wild-type and attenuated *M. bovis* strains [10]. All assays were performed in the presence of appropriate controls. Since this organism was identified on surgical biopsy specimens and as no *M. bovis* isolates were concomitantly handled in our laboratory at the time specimens were obtained, pre-analytic and in-laboratory cross contamination is highly improbable.

In 2016, the United States Centers for Disease Control and Prevention published tuberculosis treatment guidelines [18]. In these recommendations, *M. bovis* is considered as pyrazinamide mono-resistant *M. tuberculosis* and therefore justifies a 2-month combination therapy with isoniazid, rifampicin and ethambutol followed by a 7-month course of isoniazid and rifampicin. Also in 2016, Lan et al. [19] published a systematic review on the treatment of human disease due to *M. bovis*. Despite limited evidence, the authors conclude that the benefit of ethambutol as a third drug in this combination therapy is unclear and that there are no data to support a shorter than 9 months treatment regimen. We interpreted *M. bovis* as being responsible for this patient’s clinical presentation but the patient refused to complete antimycobacterial therapy despite our recommendations. One year after surgery no local relapse had occured and urine control mycobacterial culture was negative. Surgical management alone should not be considered for *M. bovis* genitourinary infections. This is reinforced by the fact that our patient most likely relapsed 6 years after an initial episode of epididymitis.

Intensive veterinary, alimentary and public health interventions have significantly reduced the burden of disease associated with *M. bovis* disease in humans. Nevertheless, infections still occur through airborne and digestive routes. This case is highly interesting since it documents isolated relapsing non-BCG *M. bovis* epididymitis without concomitant pulmonary or disseminated disease for the first time. *M. bovis* clinical and microbiological surveillance need to be maintained as this species harbors intrinsic resistance to anti-tuberculous drugs therefore requiring a specific treatment regimen.

## Abbreviations

BCG: Bacille Calmette-Guérin
PCR: polymerase chain reaction

## References

[1] Domingo M, Vidal E, Marco A (2014). Pathology of bovine tuberculosis. Res Vet Sci.

[2] O’Reilly LM, Daborn CJ (1995). The epidemiology of *Mycobacterium bovis* infections in animals and man: a review. Tuber Lung Dis.

[3] Cosivi O, Grange JM, Daborn CJ, Raviglione MC, Fujikura T, Cousins D, Robinson RA, Huchzermeyer HF, de Kantor I, Meslin FX (1998). Zoonotic tuberculosis due to *Mycobacterium bovis* in developing countries. Emerg Infect Dis.

[4] Thoen CO, LoBue PA (2007). *Mycobacterium bovis* tuberculosis: forgotten, but not gone. Lancet.

[5] Peto HM, Pratt RH, Harrington TA, LoBue PA, Armstrong LR (2009). Epidemiology of extrapulmonary tuberculosis in the United States, 1993–2006. Clin Infect Dis.

[6] Heaton ND, Hogan B, Michell M, Thompson P, Yates-Bell AJ (1989). Tuberculous epididymo-orchitis: clinical and ultrasound observations. Br J Urol.

[7] American Society for Microbiology (2016). Clinical microbiology procedures handbook.

[8] Adekambi T, Colson P, Drancourt M (2003). rpoB-based identification of nonpigmented and late-pigmenting rapidly growing mycobacteria. J Clin Microbiol.

[9] Zingue D, Flaudrops C, Drancourt M (2016). Direct matrix-assisted laser desorption ionisation time-of-flight mass spectrometry identification of mycobacteria from colonies. Eur J Clin Microbiol Infect Dis.

[10] Warren RM, Gey van Pittius NC, Barnard M, Hesseling A, Engelke E, de Kock M, Gutierrez MC, Chege GK, Victor TC, Hoal EG (2006). Differentiation of *Mycobacterium tuberculosis* complex by PCR amplification of genomic regions of difference. Int J Tuberc Lung Dis.

[11] Grange JM (2001). *Mycobacterium bovis* infection in human beings. Tuberculosis (Edinb).

[12] Courtenay O, Reilly LA, Sweeney FP, Hibberd V, Bryan S, Ul-Hassan A, Newman C, Macdonald DW, Delahay RJ, Wilson GJ (2006). Is *Mycobacterium bovis* in the environment important for the persistence of bovine tuberculosis?. Biol Lett.

[13] Evans JT, Smith EG, Banerjee A, Smith RM, Dale J, Innes JA, Hunt D, Tweddell A, Wood A, Anderson C (2007). Cluster of human tuberculosis caused by *Mycobacterium bovis*: evidence for person-to-person transmission in the UK. Lancet.

[14] Lim A, Eleuterio M, Hutter B, Murugasu-Oei B, Dick T (1999). Oxygen depletion-induced dormancy in *Mycobacterium bovis* BCG. J Bacteriol.

[15] Mateos Colino A, Sousa Escandon MA, Golpe Gomez R, Garcia Figueras R, Perez Valcarcel J, Fernandez MA (2003). Tuberculous epididymitis caused by *Mycobacterium bovis*. Arch Esp Urol.

[16] Shimura H, Ihara T, Mitsui T, Takeda M (2017). Tuberculous granuloma in the scrotal skin after intravesical Bacillus Calmette-Guerin therapy for bladder cancer: a case report. Urol Case Rep.

[17] Koizumi T, Nakanishi R, Taue R, Yamaguchi K, Nakatuji H, Kishimoto T, Izaki H, Oka N, Takahashi M, Fukumori T (2008). Case of tuberculous epididymitis caused by intravesical BCG therapy. Hinyokika Kiyo.

[18] Nahid P, Dorman SE, Alipanah N, Barry PM, Brozek JL, Cattamanchi A, Chaisson LH, Chaisson RE, Daley CL, Grzemska M (2016). Official American thoracic society/centers for disease control and prevention/infectious diseases society of America clinical practice guidelines: treatment of drug-susceptible tuberculosis. Clin Infect Dis.

[19] Lan Z, Bastos M, Menzies D (2016). Treatment of human disease due to *Mycobacterium bovis*: a systematic review. Eur Respir J.

